# Chatbots and messaging platforms in the classroom: An analysis from the teacher’s perspective

**DOI:** 10.1007/s10639-023-11703-x

**Published:** 2023-05-24

**Authors:** Juan J. Merelo, Pedro A. Castillo, Antonio M Mora, Francisco Barranco, Noorhan Abbas, Alberto Guillén, Olia Tsivitanidou

**Affiliations:** 1 Department of Computer Engineering, Automatics, and Robotics and CITIC, University of Granada, Granada, Spain; 2 Departament of Signal Theory and Telecommunications and CITIC, University of Granada, Granada, Spain; 3 Leeds University, Leeds, UK; 4 Inquirium Ltd, Nicosia, Cyprus

**Keywords:** Chatbots, Messaging platforms, Tutorship, Educational bots, Higher education

## Abstract

Messaging platforms are applications, generally mediated by an app, desktop program or the web, mainly used for synchronous communication among users. As such, they have been widely adopted officially by higher education establishments, after little or no study of their impact and perception by the teachers. We think that the introduction of these new tools and the opportunities and challenges they have needs to be studied carefully in order to adopt the model, as well as the tool, that is the most adequate for all parties involved. We already studied the perception of these tools by students, in this paper we examine the teachers’ experiences and perceptions through a survey that we validated with peers, and what they think these tools should make or serve so that it enhances students learning and helps them achieve their learning objectives. The survey has been distributed among tertiary education teachers, both in universitary and other kind of tertiary establishments, based in Spain (mainly) and Spanish-speaking countries. We have focused on collecting teachers’ preferences and opinions on the introduction of messaging platforms in their day-to-day work, as well as other services attached to them, such as chatbots. What we intend with this survey is to understand their needs and to gather information about the various educational use cases where these tools could be valuable. In addition, an analysis of how and when teachers’ opinions towards the use of these tools varies across gender, experience, and their discipline of specialization is presented. The key findings of this study highlight the factors that can contribute to the advancement of the adoption of messaging platforms and chatbots in higher education institutions to achieve the desired learning outcomes.

## Introduction

New technologies have been introduced and widely adopted into the classroom in recent years. However, in order to be successful, their application will require a certain amount of extra work and training for the teacher, as well as for the students. Training will help the two collectives involved acquire new skills and, potentially, pupils will acquire a greater level of engagement with the subject matter, which is usually the main objective. Even if several technologies are already in use inside the classroom, there is still room for adopting others.

However, the success of this adoption largely depends on the willingness and ability of teachers to embrace it, as they are the primary agents in this new educational approach. As such, gathering and assessing feedback from these users will be crucial in realizing the potential benefits of the technology.

Over the past decade, messaging apps like WhatsApp and Telegram have become increasingly popular in teaching at the secondary and higher education levels Gachago et al. (2015); Mwakapina et al. (2016); Yin (2016); Panah and Babar (2020). By creating groups at various levels (e.g. class, assignment, course), teachers and students have been able to use these apps for bilateral or group-level communication. In addition to their primary function of communication, many messaging programs also allow the creation of applications that can act as users through open application programming interfaces (APIs), which are often available through a free or freemium model.

This opens the door to the introduction of *chatbots*, that is, autonomous agents able to communicate and interact with humans (or other chatbots) in a natural way Gong (2008); Studente et al. (2020), answering question and even posing their own ones to the user, in the classroom; in this limited environment, chatbots can appear as regular users, thus being close to passing a Turing Test Moor (2003), which gives them certain characteristics that, again, can enhance student engagement. Chatbots implement different Artificial Intelligence techniques, such as natural language processing as well as, in some cases, learning; this is not a requirement, however, and in most cases they are simply applications that have a conversational interface, as opposed to a graphical user interface.

These qualities have made education one of the most promising areas for chatbot applications today Clarizia et al. (2018); Smutny and Schreiberova (2020). In this domain, they facilitate the so-called *personalized learning*, i.e. they adapt themselves to students’ pace of learning and provide online tutoring outside the classroom, which have been proved to be very effective increasing their engagement and participation in the subjects Agarwal and Wadhwa (2020). Moreover chatbots’ tutoring possibilities aid to reduce the stress of students Daniels (2016); Agarwal and Wadhwa (2020) by avoiding the face-to-face tutoring with their teacher, or asking questions in front of the class. This situation normally conducts to a silence of the student and a later contact with the teacher by e-mail, which, in turn, will mean a workload for the teacher, that could be very significant given the existing disproportion between number of students per each teacher.

Hence, the chatbot technology has a potential to mitigate this problem by providing answers to students’ questions and facilitating a dynamic and autonomous learning experience Griol et al. (2014); Kim (2020). In addition, using an automated system such as a chatbot will allow teachers to spend more time on other topics that students struggle with.

Given the potential benefits that chatbots could bring to the classroom, the focus of the unnamed European project, the main sponsor of this study, is to explore best practices and innovative use of chatbots, and to create a learning community of educators in higher education institutions. Researchers and teachers participating in this project have developed several studies. Most of these works have been focused on how messaging applications and/or chatbots are normally applied to deliver personalized learning in classrooms that occurs anytime anywhere, and promote collaborative learning experiences or group discussions Panah and Babar (2020), and boost students’ sense of belonging to their institutions Abbas et al. (2022). The majority of these studies were conducted focusing on the teachers’ perspectives, so this paper aims to provide more conclusions regarding their opinions and needs, as well as the existing challenges or opportunities that arise when adopting these technologies in classrooms. This will lead to clarify how they can positively contribute to enhancing the learning process in higher education institutions.

In this study, data collection involved two phases:

- The first phase aimed to collect feedback from students about their students’ preferences of tools/applications for chatting and messaging, how they use them in educational context, who they like to be with in the class messaging groups, and their expectations of chatbots in assisting them during their learning process. Therefore, two surveys for bachelor and master degree students at the University of Granada (Spain) were designed and answers from more than 250 students were collected. The key results from the student surveys indicate a preference for using familiar messaging applications, such as Telegram or WhatsApp. Students also expressed interest in using chatbots that can assist them with organizing their course schedules, help them access their assignment grades and facilitate searching for resources.
- The results of the first phase were fed into the second phase of the study, which focused on teachers’ opinions about using chatbots and messaging applications in classes. These results are the main focus of this paper.

Initially, a single survey was developed for this purpose and responses from 300 higher education teachers were collected and analysed; this will be detailed in Section 3. The responses’ analysis led to the preparation of a second survey for teachers and 200 responses were collected, again following the methodology that will be explained later. The questions of the second survey focused on asking about teachers’ needs, opinions and preferences of the development of future technology-enhanced tools and its potential impact on educational institutions policies. In order to evaluate these factors, this paper presents the following research questions:

- RQ1 - Do teachers use now or want to use messaging apps in their classes?
- RQ2 - Which chatbots’ features would teachers find useful for this purpose?
- RQ3 - Which kind of interaction do teachers prefer with their students?
- RQ4 - Which kind of interaction media features do teachers value the most?

We will try to respond to these research questions through the analysis of the answers provided by professors to the two aforementioned surveys. Eventually, the main research outcome of this survey should be a series of recommendations to make a successful deployment of chatbot technologies (and, in some cases, general instant messaging applications) in higher education; from the response to RQ1 and RQ4 we will try to recommend specific technologies or applications to be deployed in the classroom; the response to RQ2 and RQ3 will help us recommend chatbots features or specific platforms; and finally, from RQ3 we will also try to find best practices in the adoption of messaging platforms and their matching chatbots.

The remainder of the paper is organized as follows: first, an overview of what current research has found about the use of messaging applications, including chatbots, in the classroom is described. The methodology used in the surveys is presented in Section 3, and the results of the surveys are presented next in Section 4. Finally, we discuss these results and conclude with a series of recommendations for the successful deployment of chatbots in higher education.

## State of the art

The widespread and rapid adoption of free Mobile Instant Messaging (MIM) tools/platforms such as WhatsApp, Telegram, WeChat and Facebook Messenger stems from their simplicity, ease of use and multi-modality (i.e. video, audio, text) Tang and Hew (2017). Using these tools in higher education institutions facilitates the delivery of personalized learning that occurs anytime anywhere, and promotes collaborative learning experiences and group discussions Panah and Babar (2020).

WhatsApp is, at least in most Western countries, the most popular MIM platform. The reason is that it is the tool preferably used by educators to provide assignments’ feedback to students, support course discussions, and post learning resources in a semi-formal learning context Panah and Babar (2020). Moreover, the use of WhatsApp in higher education could enhance social presence Tang and Hew (2017) and foster trust relationships between educators and students embedded in the social learning process Gachago et al. (2015). However, this last paper also reflects the need for learners to “take ownership of the tool” and the advantages of social learning in general. At the same time, it also mentions different challenges, among which the most important is the blurring of social and academic life; indeed, there are challenges when using MIM tools that occur due to the blurring of boundaries between academic and private life. This can lead to technostress Gachago et al. (2015), difficulty in managing responsibilities, especially among mature students, and lack of privacy Tang and Hew (2017). Students’ dropout of the MIM groups, as they can leave groups at any time, can hinder their learning and undermine educators’ efforts Mwakapina et al. (2016). In addition, there is a need to set rules and norms for these MIM groups in order to maintain the safety of these online communities for students Abbas et al. (2022). However, these rules should not affect students’ ownership and control, since it is vital to advance in their learning Gachago et al. (2015) process. This is why examining the role of MIM platforms in higher education, as well as applications based on them such as chatbots, is still a challenge, and why the opinions of the teaching community towards them have to be examined, as we do in this paper.

The use of MIM platforms, although possibly valuable by itself, can be enhanced with the use of chatbots, which are conversational agents that normally dwell in synchronous conversations systems. The use of chatbots in higher education is still in its early stages Yang and Evans (2019). Nevertheless, recent studies have shown their positive impact on students’ academic performance Pérez et al. (2020) and engagement Studente et al. (2020); Abbas et al. (2022), leading to a growing interest in using this technology in the (possibly virtual) classroom. Indeed, using chatbots to collect course feedback from students in higher education improved the quality of responses given by students in their assignments, and boosted engagement levels Abbas et al. (2021). According to Roblyer et al. (2010), the use of mobile devices along with gamification strategies Yildirim (2017) can improve student motivation. In this sense, the authors in Pimmer et al. (2019) adopted a quasi-experimental, survey-based approach to report the positive impact of using instant messaging tools in boosting students’ knowledge and mitigating their feelings of isolation.

Multiple studies have been conducted to assess the effectiveness of chatbots in higher education; among them, a comprehensive paper was presented by Smutny and Schreiberova Smutny and Schreiberova (2020) reviewing 47 educational chatbots implemented in Facebook Messenger, concluding that most of these chatbots were very basic and that programmers require of more support to develop and offer tools for improving coaching methods and student learning outcomes. Pérez and collaborators Pérez et al. (2020) attempted to categorize educational chatbots attending to their purpose as service-oriented or teaching-oriented. Service-oriented chatbots include those that provide service support, such as Ask Holly Durham University (2021) and Dina Santoso et al. (2018), which respond to students’ questions about enrollment and registration. Ask L. U.^1^. answers students’ frequently asked questions about schedules, grades, tutors, and societies. LISA Dibitonto et al. (2018) and Differ Studente et al. (2020) facilitate breaking the ice between new students by introducing them to each other. Ranoliya et al. Ranoliya et al. (2017) proposed a generic chatbot for university students that is able to answer frequently asked questions. The University of Granada deployed Elvira, a chatbot embedded in its main web page Moreo et al. (2012) to perform the same task. In addition to being able to answer pre-established frequent questions, it did so from the website of the University of Granada using an inset persona, voiced by a real person, who lip-synced the answers. However, in the case of Elvira, the emphasis was on the authenticity of the speech-mouth and face gestures synchronization rather than making updating answered questions simpler or more interactive for the administrative staff, or more open to the rest of the university staff using actual messaging platforms. In most cases, the embedded search engine was able to provide more up-to-date and accurate answers than those provided by Elvira, which had to be updated by hand (and not too often). It was eventually discontinued, and its technology was not adapted to other platforms, since it did not really provide a useful service. Its service was not tied to teaching anyway; it was more related to directory queries and university-wide administrative questions, so even if it is strictly an example of a chatbot in a university setting, it is not really a chatbot that can be adopted by teachers to affect the learning outcomes, which is the focus of this paper.

Contrarily to the mentioned chatbots that are focused on administrative tasks, teaching-oriented chatbots are more sophisticated, as they set personalized learning outcomes and monitor learning progress. For instance, Fernoagă et al. (2018) reported on “eduAssistant”, a virtual teaching assistant chatbot developed on the Telegram messaging platform. In this study, the Telegram platform was chosen because it is easy to use, students are familiar with its features, and it enables them to exchange messages in different formats (text, audio and video) Fernoagă et al. (2018). In addition, Telegram can operate on all devices and operating systems. The “eduAssistant” chatbot acts as an automatic agent in the teacher-content-student relationship, providing real-time feedback loops and a personalized learning experience relevant to the students’ acquired skills and knowledge. Using this chatbot, educators can create interactive instances in their lectures where they pose questions to their students and the chatbot assists those who need further help by giving them more hints and reporting it to the educator’s dashboard Fernoagă et al. (2018). This could help educators identify students who need more attention and provide them with more educational resources relevant to their academic achievement.

Despite its (arguably) successful implementation in different higher education institutions, particularly on the fringes of educational activity rather than actual student-teacher interaction, its implementation or deployment is not trivial. Some authors, for instance Sjöström et al. (2018), have proposed a conceptual architecture for the adoption of teaching-oriented chatbots in higher education. This conceptual architecture is based on a systematic literature review of previous studies examining the design of chatbots in higher education, as well as making a content analysis of student emails and discussion forum posts from four instances of a Java programming course. The study outlined several design considerations; among them, the authors emphasized the importance of developing chatbots in platforms that students and educators are familiar with and can easily access (i.e. Facebook Messenger), which was confirmed by Hobert (2019) and Fernoagă et al. (2018). In addition, Sjöström et al. (2018) argued that conceptualizing learners’ questions could help designers integrate the appropriate types of questions that chatbots should support for different courses. Other authors, such as Coronado et al. (2018), have proposed agents that record learning materials to be provided on demand to students, while Crockett et al. (2017) reported on tutoring systems that can perform initial assessments of students’ understanding and provide learning materials to advance their understanding to the next level.

Regarding the factors for the adoption of chatbots in higher education, many studies have focused on the evaluation of technology acceptance and usability Roblyer et al. (2010); Pimmer et al. (2019). However, higher education is a special domain where, according to Hobert (2019), specific pedagogical factors such as learning success and increased motivation are more important. Therefore, to develop effective chatbots for higher education, the needs of all stakeholders (i.e. educators, students, institutions, etc) should be carefully collected and taken into consideration Sjöström et al. (2018); Tsivitanidou and Ioannou (2020). These needs include, but are not limited to, student learning success due to higher motivation, but these are a posteriori effects that cannot be assessed in advance. Both authors focus on what teachers need in terms of the latter; this paper will focus on a wider perspective, trying to determine what they are looking for in terms of general messaging technology. The classical literature Moore and Benbasat (1991) already proposes that any adoption of technology must be tuned to the user needs and experience. In this paper, we will try to find out what those are in order to propose a successful model of adoption of chatbot technology.

In line with this research, our previous work Same-Authors (2021) aimed at analyzing the expectations of students in this regard. Another paper have also analysed how the use of chatbots affected the learning outcomes of students in a Chinese class Chen et al. (2020). This paper considered conversational chatbots in a one-to-one setting, finding that learning truly benefited from it. This is, once again, an evidence of the benefit of chatbots in certain settings; however, there are some prior experiences, as well as needs, that may prevent the successful acceptance of the technology, and these are what we are trying to find out in this paper, along with what kind of features would improve its acceptance.

Besides, this work is focused on the other key actor in this challenge: educators. How they accept chatbots has been studied very recently by Chocarro et al. Cortiñas et al. (2021), who, by analyzing surveys, created a TAM that proposed a series of features that would make chatbots easier to accept, including formality of language as well as easiness and usefulness. The survey targeted primary and secondary education teachers and was also more interested in the general use of chatbots in education, not specifically in a classroom setting, seeking educational outcomes. However, their results are obviously interesting and relevant for this work.

Next, we will describe the methodology we employed to gather teachers’ perspectives.

## Methodology

This study employed a quantitative approach to address its research objectives. In order to fulfill them, we would need to collect a significant amount of data on the usage of messaging applications and chatbots by educators in universities and colleges.^2^ In order to collect this data, two online surveys were designed and developed using Google Forms in Spanish. These surveys consisted of six questions on demographic data (such as sector, gender, degrees, discipline, age, or teaching experience) followed by several multiple-choice questions allowing participants to choose multiple answers.

The **first survey** consisted of multiple-choice questions focused on the use of messaging apps in teaching practice, the type of chatbot use cases that educators would find useful for their teaching and the impact of COVID on teaching practice. These questions can be found in their entirety in the Appendix.^3^ This survey was piloted by the authors of this paper and their colleagues before using it in the study, and all feedback from this trial was incorporated into the survey. The survey link was primarily distributed via mailing lists and Telegram groups in order to reach a larger number of educators. The form targeting university teachers was sent to university in Spain (mainly in Andalucía and Galicia), and also universities in Costa Rica and Mexico. Tertiary (non-university/college) educators who received the form were mainly based in Andalucía. The survey was disseminated and responses were collected in the first quarter of 2021, during the Covid-19 pandemic, while at least in Spain, many universities were implementing mandatory virtual teaching.

A total of 282 educators responded to the survey: 193 teaching at the University (68.4%) and 89 at other tertiary education institutions (31.6%). Of those, 179 indicated their gender as male (63.5%), 98 as female (34.8%), and 5 teachers preferred not to indicate their gender (1.8%). In terms of age, the majority of participants (n=111) in this survey were 45-55 years old (39.4%), while 91 educators were 35-45 (32.3%), 46 were 25-35 (16.3%), and 34 were older than 55 (12.1%). Lastly, 84 educators had a teaching experience of 16-25 years (29.8%), 75 educators had 6-15 years of experience (26.6%), 69 educators had 0-5 years of experience (24.5%), and finally, 54 educators had more than 25 years of teaching experience (19.1%).

Responses were automatically stored in a Google Drive spreadsheet. Eventually, the results from the two forms used for the first survey were collated in a single spreadsheet. Survey questions can be found in Appendix (Section 1).

The **second survey** was designed after initial results for the first survey arrived, and pointed out necessities and experiences of educators not covered by the first survey, specifically their experience with messaging platforms and the ways they were used to interact with students. It was piloted with a group of university teaching staff attending a training course, and validated by them. The survey was then extended to the rest of responders, using the same media: Telegram groups, email, and announcements in mailing lists. The questions asked in this survey can also be found in Appendix (Section 1).

A total of 205 educators responded to the second survey: 187 graduate teachers (91.2%) and 18 student teachers (8.8%). Of those, 124 were male (60.5%), 65 female (31.7%), and 16 participants preferred not to indicate their gender (7.8%). In terms of age, the majority of the participants (n=70) were again, as in the first survey, 45-55 years old (34.1%), while 67 participants were 35-45 years old (32.7%), 42 were 25-35 (20.5%), and 26 participants were older than 55 (12.7%). In terms of teaching experience, 59 participants had a teaching experience of 16-25 years (28.8%), 51 participants had 6-15 years (24.9%), 51 had 0-5 years of experience (24.9%), and 44 participants had more than 25 years of teaching experience (21.5%).

### Survey validation

To avoid any kind of bias and to ensure the quality of the questionnaire, a panel of experts was consulted. The panel board evaluated three aspects for each item of the questionnaire: Clarity, Importance, and Adequateness. Clarity was measured as an answer to the question: “Is the item well presented and without ambiguity?”^4^ with two choices: “Yes” or “No”. Both Importance and Adequateness were measured on a Likert scale from 1 to 4 (1 being low and 4 being high).

The panel board was composed of 7 volunteer experts that have wide experience as educators and researchers. The results obtained are summarized in Table 1.

As validation shows, all experts agree without any doubt about the clarity of each question. Furthermore, there is a large consensus regarding the Importance and Adequateness of all eight questions in the questionnaire.

**Table 1:** Validation results from the panel board (7 experts) for each question in the research questionnaire

Questions	Clarity (Yes/No)	Avg. Importance	Avg. Adequateness
Q1	Yes (100%)	3,571 (0,53)	3,571 (0,53)
Q2	Yes (100%)	3,714 (0,49)	3,571 (0,53)
Q3	Yes (100%)	3,857 (0,38)	3,857 (0,38)
Q4	Yes (100%)	3,571 (0,79)	3,857 (0,38)
Q5	Yes (100%)	3,714 (0,49)	3,714 (0,49)
Q6	Yes (100%)	3,714 (0,49)	3,857 (0,38)
Q7	Yes (100%)	3,714 (0,49)	3,714 (0,49)
Q8	Yes (100%)	4 (0)	4,000 (0)

The range of values for Importance and Adequateness is from 1 to 4 (higher is better)

In the next section, we present the results and analyse them.

## Results and analysis

We collected responses from 282 teachers from Spain and Spanish-speaking countries for *survey1* and 205 for *survey2*. The two forms were open for approximately the same amount of time, around two months. Most responders were university teachers, although about 32% of them in *survey1* and 8% (in *survey2* are from non-universitary tertiary education teachers. With respect to gender, 61% of teachers were male and 32% female, while approximately 5% chose not to disclose their gender. Finally, the responses are more or less equally distributed according to the teacher’s years of experience, showing 24% of responses from teachers with 5 or less years of experience, 26% for 6-15 years of experience, 30% for 16-25, and 20% for teachers with more than 25 years of experience.

We will try to assess whether we found the answers to the four research questions next, by analyzing the responses to the different survey questions.

### RQ1 - Are teachers already using messaging apps in their classes?

Group messaging apps have become a powerful tool for communicating with students. Also, many of them have embedded chatbots to enhance the learning process. After an initial analysis of the answers to the first survey, we realized that there were some prior issues mainly related to the adoption of a technology, i.e. chatbots, that generally piggybacks on another, i.e. messaging applications. Generally, in a technology adoption model, perceived ease of use and perceived usefulness are essential. But it will be very difficult for teachers to find *chatbots* easy to use if they do not already consider or simply use, the messaging tools to which they are attached.

This is why, in order to answer RQ1, teachers were queried about whether they use messaging apps in their classes, specifically Telegram, WhatsApp, Slack (an application used mainly in IT departments and software development), or any other messaging app. They were also asked if they use messaging apps provided by their academic institution (see Table 2). Overall, the majority of teachers responded that they do use messaging apps in their classrooms. The most common responses were messaging apps (or, more probably, messaging solutions) provided by their academic institutions (n=159, 56%) and WhatsApp (n=124, 44.0%). Only 19 teachers (6.7%) replied that they do not use any messaging app in their class.

**Table 2:** Use of messaging apps to assist the learning process

Messaging App	Yes		No	
	Frequency	%	Frequency	%
Telegram	62	22.0	220	78.0
WhatsApp	124	44.0	158	56.0
Slack	15	5.3	267	94.7
Other	60	21.3	222	78.7
Provided by the Academic				
Institution	159	56.4	123	43.6
None	19	6.7	263	93.3

**Fig. 1:**
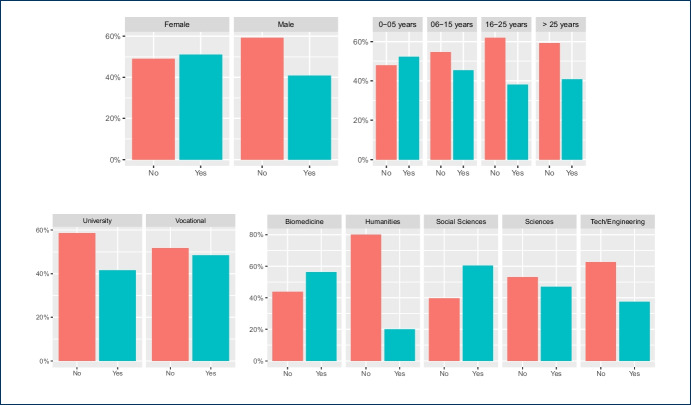
Use of messaging platforms either external or provided by the teachers’ academic institutions in class: distributions per gender, years of experience in education, university or vocational education, and discipline

**Fig. 2:**
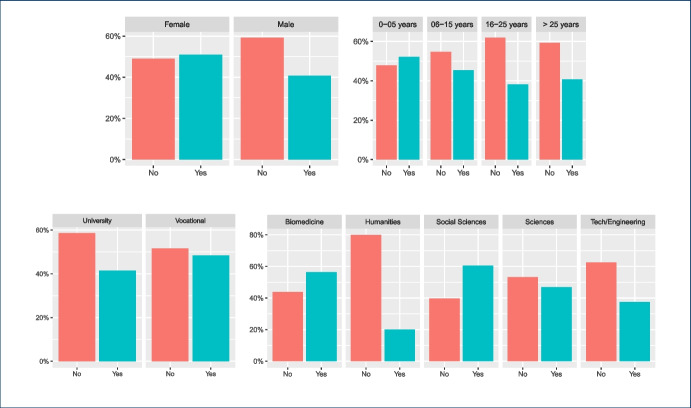
Use of Whatsapp in class: distributions per gender, years of experience in education, university or vocational education, and discipline

**Fig. 3:**
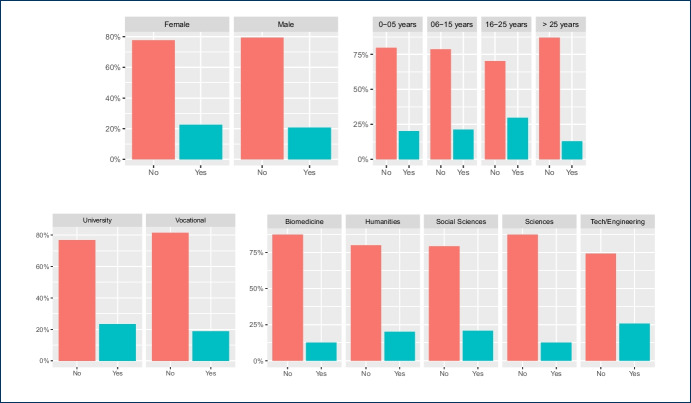
Use of Telegram in class: distributions per gender, years of experience in education, university or vocational education, and discipline

Results in Figs. 1, 2, 3 do not show significant differences in the use of instant messaging apps between teachers from universities and vocational education institutions. In general, most teachers prefer messaging apps provided by their own institutions (we also show the results for the two most popular messaging app platforms: WhatsApp and Telegram). With respect to specific disciplines, Engineering and Technology teachers are more active in their use, but it is also remarkable the number of teachers from Humanities who answered they used these apps in their classes (around 60% use the apps provided by their institutions).

Although no significant differences were found regarding gender, female teachers answered they use instant messaging apps more than male teachers (about 10% more). Also, teachers in vocational Education use WhatsApp more than university teachers. Regarding the distribution of the use of messaging apps per age, there are no significant differences for WhatsApp and apps provided by their own academic institutions. However, younger teachers also use Telegram with more than 25% responding that they do, a percentage that falls to about 10% for teachers that are 55 or older. One interesting result is that about 65% of teachers with more than 25 years of experience use the platforms provided by their institutions while the percentage goes down to less than 50% for teachers with 6-15 years of experience.

**Fig. 4:**
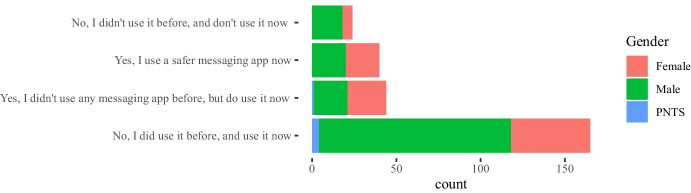
Total count of responses for the use of messaging apps after the COVID-19 pandemic grouped by gender (PNTS stands for Prefer Not To Say)

**Fig. 5:**
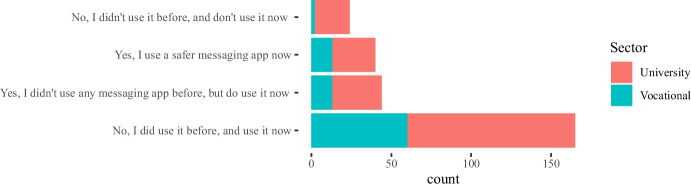
Total count of responses for the use of messaging apps after the COVID-19 pandemic grouped by sector

**Fig. 6:**
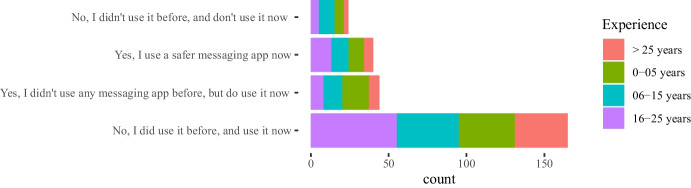
Total count of responses for the use of messaging apps after the COVID-19 pandemic grouped by years of experience in teaching

**Fig. 7:**
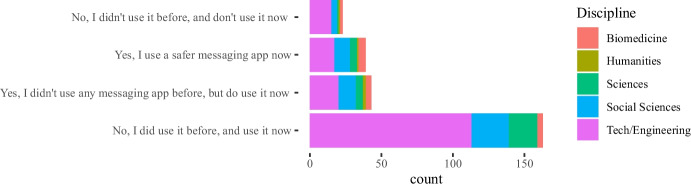
Total count of responses for the use of messaging apps after the COVID-19 pandemic grouped by discipline

Some of the questions in the survey were focused on the impact of the COVID-19 pandemic between the 2020 and 2021 academic years on the teachers’ attitudes towards the use of instant messaging apps in their classes. Our main intention in this case was to assess whether a crisis will bring about a change in the use of these tools. The 282 answers are summarized in Figs. 4, 5, 6, 7, showing that about 77% of teachers already used these tools before the pandemic and kept using them during the pandemic lockdowns that forced students and educators to use remote education. Additionally, approximately 15% of them switched their messaging app for one that offered a safer interaction with their students. According to the responses, an additional 16% started using messaging apps during the pandemic for the first time in their classes.

**Table 3:** Sector* post-COVID-19 changes cross-tabulation

		Sector		Total
		University (f)	Vocational (f)	
Post covid changes	Yes, I use a safer messaging app now	27	13	40
	Yes, I didn’t use any messaging app before, but do use it now	31	13	44
	No, I did use it before and use it now	105	60	165
	No, I didn’t use it before and don’t use it now	22	2	24
Total		193	89	282

Furthermore, a chi-square test of independence was performed in order to examine the relationship between instructors’ discipline, sector, and gender and any potential changes that occurred to the use of messaging apps due to the COVID-19 pandemic. The relationship between the latter variable and the instructors’ sector was significant, $X^{2}_{(4, N = 282)} = 9.598$, $p =0.048$. Table 3 shows the frequencies that are cross-tabulated versus changes undertaken after the COVID-19 pandemic. This finding suggests that how teachers used messaging apps during the pandemic, specifically whether they changed their habits for teaching purposes, was related to the sector (university vs. vocational) in which they were working.

The majority of the teachers (n=165) mentioned that no changes in their habits occurred due to the shift to remote teaching due to the lock-down measures taken in their countries, as the use of messaging apps was part of their teaching practices and remained the same. Out of those 165 responders, 105 teachers come from the university and 60 teachers from the non-universitary tertiary sector. The relationships between changes in the use of messaging apps, due to the COVID-19 pandemic, and the instructors’ discipline, $X^{2}_{(24, N = 282)} = 44.856$, $p =0.006$, as well as the gender, $X^{2}_{(8, N = 282)} = 16.249$, p= 0.039 were also significant. This finding indicates that how teachers responded to the use of messaging apps during the pandemic was also related to their gender and discipline. In fact, from the majority of the teachers who did not change their habits in this respect (n=165), most of them are males (n=114) and come from the technology (n=60) and engineering (n=53) disciplines (see Tables 4, 5).

**Table 4:** Gender * post-COVID-19 changes crosstabulation

		Gender			Total
		Male	Female	PNTS	
Post-covid changes	Yes, I use a safer messaging app now	20	20	0	40
	Yes, I didn’t use any messaging app before, but do use it now	20	23	1	44
	No, I did use it before and use it now	114	47	4	165
	No, I didn’t use it before and don’t use it now	18	6	0	24
	Other	7	2	0	9
Total		179	98	5	282

**Table 5:** Discipline * post-COVID-19 changes crosstabulation

		Disciplines							Total
		Engineering(f)	Social Sciences (f)	Sciences (f)	Biomedicine (f)	Humanities(f)	Technology(f)	Other(f)	
Post-covid changes	Yes, I use a safer messaging app now	7	11	5	5	1	10	1	40
	Yes, I didn’t use any messaging app before, but do use it now	13	12	5	4	2	7	1	44
	No, I did use it before and use it now	60	26	20	4	0	53	2	165
	No, I didn’t use it before and don’t use it now	14	4	1	2	1	1	1	24
	Other	5	0	1	1	1	1	0	9
Total		99	53	32	16	5	72	5	282

**Fig. 8:**
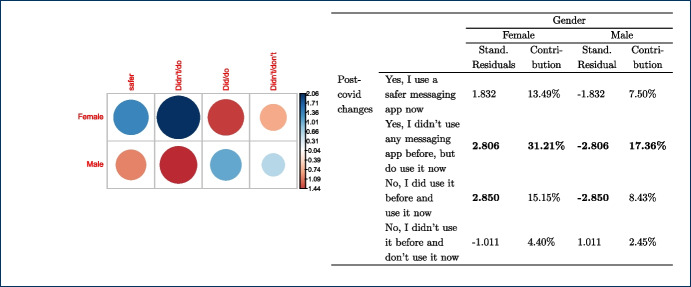
Analysis for gender and post-COVID-19 changes: left) Pearson’s residuals for the Chi-square test; right) Gender*post-COVID-19 changes crosstabulation, including standardized residuals Agresti (2013) (also adjusted standardized residuals) and contribution percentage to the total Chi-square test of each cell

**Fig. 9:**
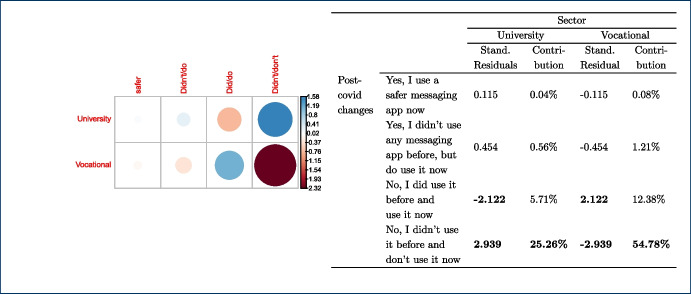
Analysis for sector and post-COVID-19 changes: left) Pearson’s residuals for the Chi-square test; right) Sector*post-COVID-19 changes crosstabulation, including standardized residuals Agresti (2013) (also adjusted standardized residuals) and contribution percentage to the total Chi-square test of each cell

**Fig. 10:**
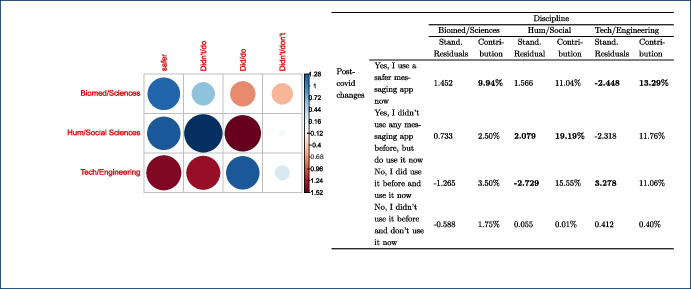
Analysis for discipline and post-COVID-19 changes: left) Pearson’s residuals for the Chi-square test; right) Discipline*post-COVID-19 changes cross-tabulation, including standardized residuals Agresti (2013) (also adjusted standardized residuals) and contribution percentage to the total Chi-square test of each cell

Finally, we took a closer look at the post-COVID-19 changes and the variables gender, sector, and discipline for which the chi-square test is statistically significant as shown in Figs. 8, 9, 10 respectively. Each figure shows on the left, a graph that represents the Pearson’s residuals of the chi-square test results and a table on the right part that shows the contribution of each cell to the test. In the Pearson’s residual graphs, the responses to the question about the use of messaging apps were shortened for the sake of clarity according to: 1) *Did/do* stands for *Yes, I did use it before and do use it now*; 2) *Didn’t/do* for *Yes, I didn’t use any messaging app before, but do use it now*; 3) *safer* for *Yes, I use a safer messaging app now*; 4) *Didn’t/Don’t* for *No, I didn’t use it before and don’t use it now*.

As mentioned, the graphs on the left-hand side represent the Pearson’s residuals, showing the difference between the observed and the expected values for each cell. Thus, large residuals indicate that variables are not truly independent. In our case, blue shows positive contributions and red negative contributions to the test. Additionally, the saturation shows how large the contribution is in contrast to the expected value by chance. Tables show on the right hand side adjusted standardized residuals that according to Agresti (2013), if greater than +/-2 for cases with few cells, indicate lack of fit of $H_0$ (in boldface in our table). Complementarily, the table also shows the percentage of contribution to the test of each cell (the highest percentage per column also in boldface).

Regarding gender in Fig. 8, the results show a high positive contribution from female educators that did not use a messaging app before the COVID-19 pandemic but do use it now; approximately 31% of the test results are explained by this cell. In contrast, male educators that responded in the same way were less than expected (negative contribution that explains 17% of the test results). Also, it is important to highlight the female educators that continued to use these apps before and after the pandemic and correspondingly the negative contribution of male educators to the same case (less than expected by chance).

With respect to the sector, results in Fig. 9 show a positive contribution from university educators being reluctant to use any messaging apps before or after the pandemic: the positive contribution of their responses explain 25% of the test results with an adjusted residual of 2.94. On the contrary, vocational teachers were more open to it, showing a negative Pearson’s residual. Also, we find larger values than expected in vocational teachers that continued to use these apps after the pandemic.

Finally, statistical significance was found for discipline vs. post-COVID-19 changes. Regarding disciplines (see Fig. 10), the number of educators from Humanities and Social Sciences that started using messaging apps after the pandemic is larger than expected and these number of responses explains about 19% of the chi-square test results. Also, the number of educators from Technology or Engineering that started using safer alternatives^5^ are less than expected (compared to the educators from other disciplines). Bear in mind that some disciplines were merged to avoid very low number of responses for some of the cells.

### RQ2 - Which kind of chatbots would teachers find useful in their classes?

For answering RQ2, teachers were provided with a list of different potential chatbot functionalities (use cases) and were requested to indicate whether each given use case would be useful in their classes. The findings are summarized in Table 6.

**Table 6:** Perceived useful chatbot use cases

Chatbot use cases	Yes		No	
	Frequency	%	Frequency	%
Answering to students’ FAQs	148	52.5	134	47.5
Assigning student grades	113	40.1	169	59.9
Facilitating agenda information	171	60.6	111	39.4
Sharing class materials	136	48.2	146	51.8
Others	25	8.9	257	91.1

**Table 7:** Agenda use case * FAQs use case crosstabulation

		FAQS		Total
		Yes	No	
Agenda	Yes	103	68	171
	No	45	66	111
Total		148	134	282

**Fig. 11:**
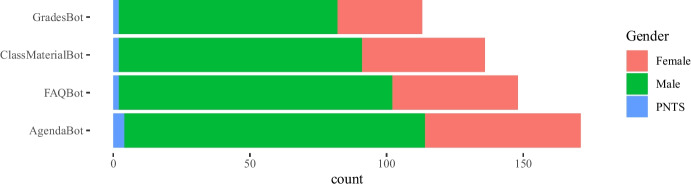
Count of types of chatbots for class perceived as the most useful for teachers grouped by gender (PNTS stands for Prefer Not To Say)

**Fig. 12:**
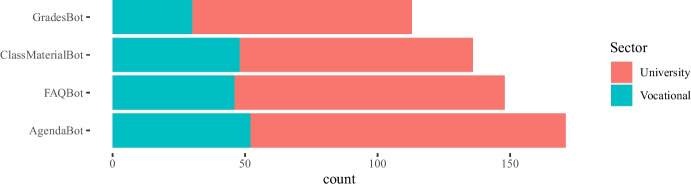
Count of types of chatbots for class perceived as the most useful for teachers grouped by sector

**Fig. 13:**
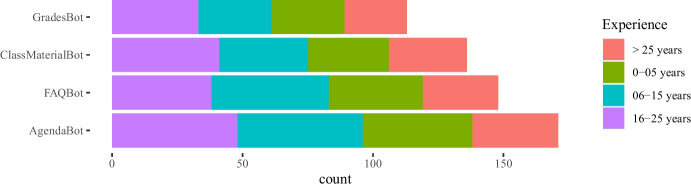
Count of types of chatbots for class perceived as the most useful for teachers grouped by experience

The most beneficial use case for chatbots in higher education and vocational training is their ability to facilitate agenda formation and communication (171 positive responses, 60.6%), followed by the FAQs use case (148 positive responses, 52.5%), and the sharing class material use case (136; 48.2%). A chi-square test of independence was performed to examine the relationship among participants’ preferences for particular chatbot use cases. Out of the 171 teachers who considered useful the use of chatbots for agenda preparation in the class, 103 also consider useful chatbots’ use for FAQs. The relationship between agenda and the FAQs use case was significant, $X^{2}_{(1, N = 282)} = 10.467$, $p =0.001$. The frequencies cross-tabulated are given in Table 7.

**Fig. 14:**
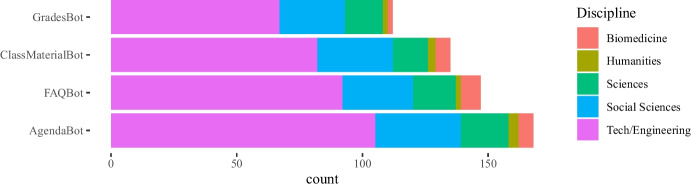
Count of types of chatbots for class perceived as the most useful for teachers grouped by discipline

Answers to these questions are plotted in Figs. 11, 12, 13, 14 grouped by gender, sector, years of experience in education, and discipline respectively. As it was a multiple-choice question, the counts are over the total number of educators who answered.

### RQ3 - Which kind of interaction do teachers prefer with their students?

Since chatbots are intended to mediate or help in this interaction, it is essential to understand the kind of interaction that teachers prefer. Chatbots should address those modes, and not others. In order to find out these modes, and thus answering RQ3, teachers were provided with a list of different kinds of interactions that may take place among students and between students and their teacher, when using messaging apps. The findings are summarized in Table 8.

**Table 8:** Kind of interactions preferred

Kind of interactions	Yes		No	
	Frequency	%	Frequency	%
Chat interactions among students in the same course	166	59.1	115	40.9
Chat interactions among students, teachers of the School/Faculty	19	6.7	266	94.3
Chat interactions among students of the same study year and teachers	58	20.5	224	79.4
Teacher not being part of the interaction	81	28.7	201	71.3

**Fig. 15:**
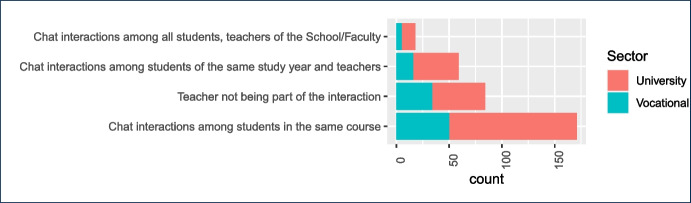
Distribution of teachers’ preferences for the chat groups with their students: from groups only with their students from a specific course to groups with greater social interaction with all students in their School or Faculty

As it can be seen by the answers to the first and last questions, in general teachers do not want to participate in a chat group with their students. They either want to simply leave the students alone in their own chat group, or otherwise they prefer not to be part of that interaction. In general, that is going to be the case no matter what; it is well known that students organize their own chat groups with many (and not always conveyable) intentions, so teachers do not want to take any part in these informal or non institutionally-supported chat groups. Overwhelmingly, they do not want to participate in this kind of chat groups with students, but even less so if it includes the rest of the faculty.

**Table 9:** Interaction media features valued by teachers

Interaction features	Yes		No	
	Freq.	%	Freq.	%
Analytics	108	52.7	97	47.3
Connectivity	119	58.0	86	42.0
Familiarity	121	59.0	84	41.0
Hidden Phone Number	113	55.1	92	44.9
Horizontally	134	65.4	71	34.6
Official formation	42	20.5	163	79.5
Pluggability	65	31.7	140	68.3
Sustainability	157	76.6	48	23.4
Unidirectionality	27	13.2	178	86.8
Officiality	150	73.2	55	26.8
Synchrony	45	22.0	160	78.0
Other	5	2.4	200	97.6

Details about each of these features can be read in Appendix (Section 1, second survey)

**Fig. 16:**
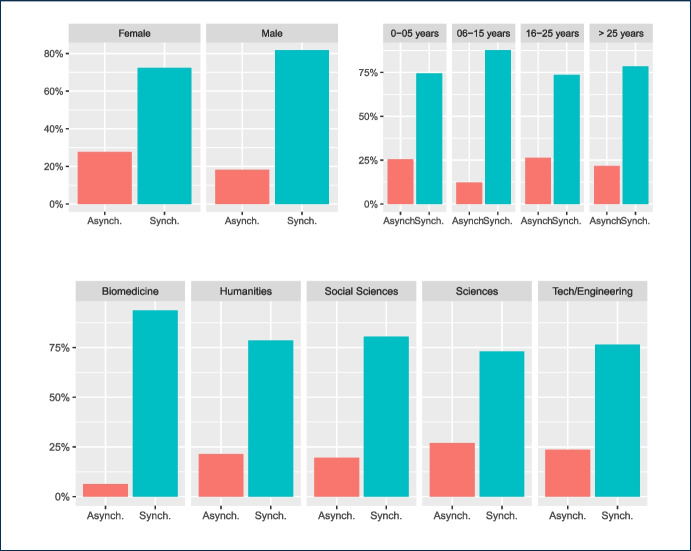
Results of teachers’ communication preferences: synchronous or asynchronous interaction with students (grouped by gender, years of experience, and discipline)

Then, the answer to this research question is that in general, teachers do *not* want to have interaction with students in a chat group. To a certain point, this would seem to contradict results Gachago et al. (2015), although this might be due to cultural attitudes, or other factors such as the average size of classes. It does confirm, however, that the challenges cited in that study, and possibly others, are an obstacle to the adoption of mobile (and other) instant messaging among the community of surveyed teachers.

Regarding the social factor of chat groups that teachers use in class, according to Fig. 15 the vast majority prefer small groups only with their students from the same course. These are more focused groups with specific goals and dedicated to the course organization and its tasks, and from the pedagogic point of view it also seems more adequate to improve the learning process. Interestingly, and as a cross-check of the answers above, about 30% of teachers consider they should not be part of the chat group. This might seem to contradict the results of the other survey, but in fact, being a result of different surveys, to a certain point affirms the same thing: there is a great amount of teachers that would be against being in a chat group with students. However, students will still need to get the services that the university, through chatbots, provide, thus opening the door for deployment of chatbots without the intervention of their professors, simply tapping university provided services Bernier et al. (2002). Whether that is a slim majority or not, that is open to debate (and would probably need a more focused survey). However, it is clear that forcing teachers to create chat groups with students and participate in them would create a certain amount of resistance. Also, only university teachers find interesting a group with all the students and teachers in their own Faculty or School. The lack of teachers from vocational education here may be the consequence of using specific language such as “Faculty”. Moreover, the fact that many universities and schools already use these groups for administrative and social interaction (e.g. Dibitonto et al. (2018)) might be the reason for the low percentage of teachers that chose this response.

### RQ4 - Which kind of interaction media features do teachers value the most?

As show in Table 9, the bulk of responders expressed their wish to use a sustainable and official application, i.e. both being approved or provided by their educational institution, and also maintained by the technical staff of the institution instead of giving this responsibility/task to the teacher. In addition, it is very important for teachers that used the tools that all the members of the class communicate, including themselves; as well as using tools that are already known or used in everyday tasks, e.g. Telegram or WhatsApp.

Figure 16 shows also a general preference for synchronous communication with students.

A chi-square test of independence was performed to examine the potential relationship among the various features. The statistically significant correlations are given in Table 10.

**Fig. 17:**
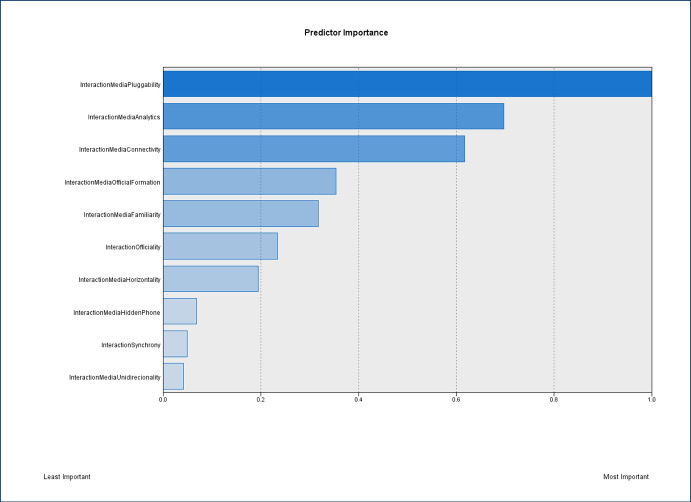
Two-step cluster analysis for interaction media features: predictor importance

Finally, a two-step cluster analysis was conducted, with a log-likelihood distance measure adopted, to explore how the use cases could be grouped based on their preferences of specific interaction media features, and which features have larger predictor importance for the clustering (see Fig. 17). Two clusters resulted from the analysis: cluster 1 (n=78, 60.9%) is formed with instructors who did not value interaction media pluggability, connectivity, and official formation, but valued interaction media analytics, familiarity, and support by their institution; cluster 2 (n=50, 39.1%) groups instructors that did not value media analytics, official formation, and familiarity, but valued pluggability, connectivity, and support by their institution. Interaction media pluggability appears to have the most important predictor importance in the clustering of cases (predictor importance = 1.0), whilst media unidirectionality was the least important factor (predictor importance = 0.04). The cluster quality is fair, but not a good one (as indicated by the silhouette measure of cohesion and separation).

**Table 10:** Interaction media features analysis ($$^{***}p<0.001$$, $$^{**}p<0.01$$, $$^{*}p<0.05$$)

	Connectivity	Familiarity	Horizontality	Official formation	Pluggability	Sustainability	Unidirectionality	Officiality
Analytics	$$12.172^{***}$$	$$4.855^{*}$$	ns	$$4.144^{*}$$	$$12.490^{***}$$	$$4.314^{*}$$	ns	ns
Connectivity	-	ns	$$5.971^{***}$$	ns	$$16.286^{***}$$	ns	ns	ns
Hidden phone			ns	$5.678^{*}$	$4.683^{*}$	ns	$4.515^{*}$	ns
Horizontality		-	ns	ns	ns	ns	ns	$5.453^{*}$
Official formation				-	$$8.163^{**}$$	ns	ns	ns

Columns and rows with no significant interaction have been suppressed for clarity. Every cell value represents $X^{2}_{(1, N = 205)}$. Details about each feature can be found in Appendix

## Discussion and implications

Our initial intention in the design of these surveys was to probe the opinions of tertiary education teachers in the introduction of chatbot technologies in class. We wanted to find the answer to four research questions, of which the first was to check whether teachers already used some kind of messaging application, and the one they preferred. The availability of certain technologies for implementing chatbots may vary based on the outcome. In this sense, the design of the survey introduced some ambiguity on what we really consider “messaging technology”, that was anyway validated in the piloting phase of the survey, and eventually resolved by the answers. Apparently, anything that sends messages (even if they are not instant messages or have no dedicated application) was the concept in the minds of the surveyed. In this case, we would like to add a clarification. While we had in mind, as indicated by the possible answers, instant messaging applications when we elaborated this question, we included these institution-provided applications mainly for completion. But it should be noted that, in most cases, they are not *instant* messaging apps in the way Telegram or Facebook Messenger are; they are messaging facilities provided by learning management systems such as Moodle or Blackboard. So the answer to this question must be understood in two different ways: their preference for the *provider* of the technology (the university itself, any company) as well as the *type* of technology (instant, app-based messaging systems vs. web- or email-based messaging systems integrated in another learning management system).

This inclusion of university-provided messaging applications in the survey might explain why there are so few teachers, just 6.7%, that answered they did not use any messaging application. In general, universities provide official channels to send (or simply make available to) students academic information such as the exam schedule or the grades. The statistical mode is to use that kind of messaging application. Some other teaching staff might not use it, but use some extraofficial or opt-in channels such as the others included in the survey; in the end, between these official and extra-official channels, most people cover their needs to communicate with students. In general, this matches the subjective experience of the authors of this paper.

Furthermore, the answers to the survey question regarding COVID-19 highlights the significant challenge of altering behavior, even during a major crisis. Very few responders actually changed their habits, although comparing women vs. men as well as those teaching social sciences vs. the rest of the disciplines, there were some significant differences. We do not have a clear understanding of the reasons behind this behavior; however, it appears that the perception and adoption of technologies in a major crisis may vary based on an individual’s background and gender.

One of the most interesting questions arising from the analysis of the results, and that should require further study, is: Are teachers really aware of the possibilities of chatbots in the classroom? The survey only listed a few functionalities for the chatbots, and just a few subjects selected the option “Others”. This may suggest that most teachers did not really understand at this point the actual potential of chatbots, since the responses listed were by no means exhaustive. Had the responders been more acquainted with the possibilities of this technology, this “Other” response would possibly have shown up many more times. Another perspective is that, without clear guidelines on how to utilize a tool, educators may not have the capacity or energy to consider innovative uses; thus, they may have assumed that it is not their responsibility to explore new possibilities with existing or new technologies.

The reluctance to adopt chatbots in class might have an explanation: We can speculate that teaching staff’s opinions are influenced by negative experiences with chatbots used in customer support, which is what most people are likely to have experienced. Further study may be needed to confirm this, but it is important to keep it in mind when introducing chatbot technologies in the classroom. To potentially change this existing perception, it will be necessary to make chatbots as simple and satisfying to use as possible.

Regarding gender grouping, both genders agree in the possible uses of chatbots, so it seems not relevant at all. However, when we look at the plot containing the sector differentiation, it is interesting to note that university teaching staff are more likely to use chatbots for grades and FAQs compared to teaching staff involved in other tertiary education institutions. This appears logical, as the number of personal interactions with students in certain classes at the university is smaller^6^, thus an automated grade notification tool for continuous evaluation would be more beneficial. Furthermore, the high student-to-teacher ratios that university educators often face could be another reason why a higher number of them are willing to use chatbots for FAQs, as this could reduce misunderstandings and email (or face to face tutoring) overload from students.

The results, when grouping by teaching experience, confirm that experienced teachers are just as open to change and willing to embrace new technological challenges as younger educators. From this concrete plot it is interesting as well to observe the fact that very few young teachers are willing to use chatbots for other uses. This reinforces the hypothesis thrown at the beginning of this section that younger teachers have less experience to consider new ways of using chatbots in their daily work.

According to the responses presented above, teachers’ reactions are evenly split between positive and negative in relation to the media features: analytics, connectivity, and hidden phone number. This means that none of these features is a deciding factor in their choice of technology. However, for features such as familiarity, horizontality, sustainability, and officiality the majority provided a positive response, designating their preference to these features. On the other hand, the majority of teachers do not seem to see the media features of official certification, pluggability, unidirectionality, and synchrony as valuable in messaging apps.

From a qualitative point of view, these results show consensus in a few key aspects:

- Teaching staff wants to have support from their institutions. One of the main reasons why this is a factor might be the strict European data regulations on data protection; it is very likely that they also want to avoid the overhead required to sign up new students every year and other maintenance tasks. In general, additional support with specific training and access to IT help desk for specific features enhance the possibility of a successful adoption that improves the learning outcomes. This is supported by the result of the two surveys: they prefer whatever “messaging” application provided by as well as the fact that this application is the “official” for the university, as shown in Table 9.
- Teachers require sustainability. The primary rationale is that the implementation of changes is costly in terms of effort and prone to mistakes during the initial years of deployment of any new technology. Therefore, it is logical that if chatbots or messaging platforms are to be introduced, teaching staff want this change to be as permanent, and long-term, as possible.
- “Keep it Simple”. Media interaction requirements are simple and only 2.4% of educators are requesting more features. This could be a sign of technological burnout produced by the previous pandemic courses, when the use of computers significantly increased; however, simplicity is always a feature that people want to have in any technology they adopt.

The analysis of the answers to questions related to RQ4 seems to indicate that teaching staff prefer to share an interaction space with their students. As shown in Tables 8 and 9, the majority of responders indicated that they *do not* want unidirectional communication, and that they prefer *horizontal* interaction. This point is reinforced by results on the kind of chatbots they preferred: most of them indicated FAQ bots, which might indicate a need to offload part of the burden of answering every single question posed by their students. In a horizontal setting, other students would answer if the teacher does not do it immediately. This might also be the reason why the possibility of using tools from which analytics can be extracted is also valued by teachers: in that way, the student activity in the chat tool, answering questions, or their regularity can all be assessed as part of the process of achieving learning objectives.

In our survey, we could not find an answer about the right moment for the introduction of chatbot technologies. The response to questions regarding the change of behavior during and after the first stay-at-home stage of the pandemic do not suggest that a major (or minor, such as new higher education laws^7^ or the introduction of new degrees) crisis could be an opportunity to introduce new technologies. The reason being that it does not bring major changes in attitude. Although changes driven by external forces do offer the chance of piloting new technologies, they do not seem to bring internal changes in educators’ attitudes (which, after all, is bound to be the same). In absence of a clear answer in this direction, the right moment to introduce new technologies is always when the IT and managing staff is ready to support it (since, as we have seen before, “official” support is one of the factors that is most valued by teachers).

## Conclusions

The key findings of this study shed light on educators’ preferences for using messaging applications supported by their institutions. The literature on technology adoption often highlights the importance of users’ perceptions of the technology’s usefulness and ease of use for its successful implementation and utilization. However, in the context of higher education, institutions also play a crucial role in integrating these tools into their educational systems. This does not only improve the uptake of these applications, but also shapes the social and educational experiences of students. To achieve this successful integration, institutions should ensure that these messaging applications are (in Europe) GDPR- (General Data Protection Regulation) compliant to protect students’ data and provide IT support to all stakeholders who use the applications. In summary, a technology adoption strategy for instant messaging, including chatbots, implies an institutional adoption strategy first and foremost.

Comparing these results with those obtained from student surveys in Same-Authors (2021), we now have a clear vision of the differences between teachers’ and students’ points of view and intentions when using the messaging applications in higher education. Teachers are more likely to adopt technologies that are supported by their institutions. This may be driven by a desire to ensure that their universities monitor and support their efforts to assist students during the learning process, although another reason could simply be their familiarity with the technological services provided by their institutions. Meanwhile, students use non-institutional messaging applications to create informal discussion groups with their peers. It is worth noting that peer support and collaboration are inseparable from learning Timmis (2012) and its presence correlates positively with higher retention rate in higher education O’Boyle (2014). Therefore, both perspectives are complementary and play different roles in promoting the learning process. However, there is a certain degree of incompatibility that would hinder the process of adoption of a single messaging service that fits the need of both collectives, or would favor the adoption of semi-informal solutions that would rely on a popular platform (such as WhatsApp or Telegram) enhanced with institutional agreements with their providers, as well as locally created and supported chatbots.

As with many other methodologies for assessing technology acceptance, the survey results suggest that the introduction of simple, and institution-supported instant messaging and chatbot technologies would increase the perceived usefulness (which is one of the key metrics in technology acceptance models). Given that most institutions already have some *virtual campus* or learning management system, adding some easy automation or connection to personal instant messaging tools could help introduce these new technologies to the learning community.

Based on the discussion of this study, we propose here a potential process for introducing technology that would need to be piloted in order to determine its value and its relationship to improved learning outcomes and higher teaching staff satisfaction.

Initially, institutions should introduce messaging automation tools attached to some, or several, messaging platforms that are already popular among the university community, that would help with bureaucratic or repetitive tasks, such as answering frequently asked questions or providing information on class or assignment deadlines. These chatbots will pave the way for more complex ones that will have a greater impact on learning outcomes, such as chatbots that help students integrate with other students in class or identify and address learning problems in individual students or groups of them. These chatbots should also be accompanied by analytics on student interaction and possibly some natural language processing (in vernacular language) to help assess the general mood of the class and how different materials or external factors affect it. These tools would have to be attached to a learning analytics platform, which in turn, would be part of an institutional learning management system.

The introduction of chatbots and chatbot technology will be helped by the fact that no discernible differences are found in the survey between different demographic groups. Even though chatbots do have some potential for personalization or customization based on the class material, student, and teaching staff, the institutional introduction of the technology can be done in a general way and for all disciplines, types or degrees, teaching experience, and gender.

Overall, the general feeling that transpires from the survey is that it is essential for any institution to take into account stakeholders’ opinions when introducing chatbots. This is true almost across the board for any new technology, but in the case of chatbots (and instant messaging applications) their immediacy and the fact that they can invade what we could call the private sphere makes this even more necessary.

One of the questions in the **second survey**, which asked about the messaging applications used by tertiary education teachers, opens a new line of inquiry about what educators *perceive* as such, and how it is used. Namely, the responses indicated that teachers considered messaging applications not only traditional chats or instant messaging apps such as WhatsApp or Microsoft Teams, but also any means provided by their university for communicating with students, such as a feature of the cmapus-wide learning management system that emails grades to students. This suggests that teachers have a need for communication with students, which is mostly unidirectional, and that it does not matter as much how that need is met. However, this will need careful consideration, including how it ties with the automation of the learning experience provided by chatbots.

Our analysis of the survey results can inform several potential avenues for future research. For instance, the rollout of extensive videoconferencing and virtual teaching solutions that the COVID-19 pandemic has brought has also taught us a series of lessons: it increases isolation and decreases the amount of synchronous contact that happens in the fringes of the classroom (e.g. teaching staff offices, or before and after class). A future line of research could focus on these issues and explore how chatbot technology could address them.

Finally, the full extent of chatbot technology is not really examined in these surveys. They can be connected to natural language processing engines with sentiment analysis as well as other analytics. Examining the mood of the class such as responses to new materials, assignments, exams or external events will help any student-centered teaching strategy, which will also help students (and teachers) achieve their learning objectives. This line of work, however, would require the introduction of some pilot study combined with initial opinion assessments from students and is thus left as future work.

## Figures and Tables

**Fig. 18:**
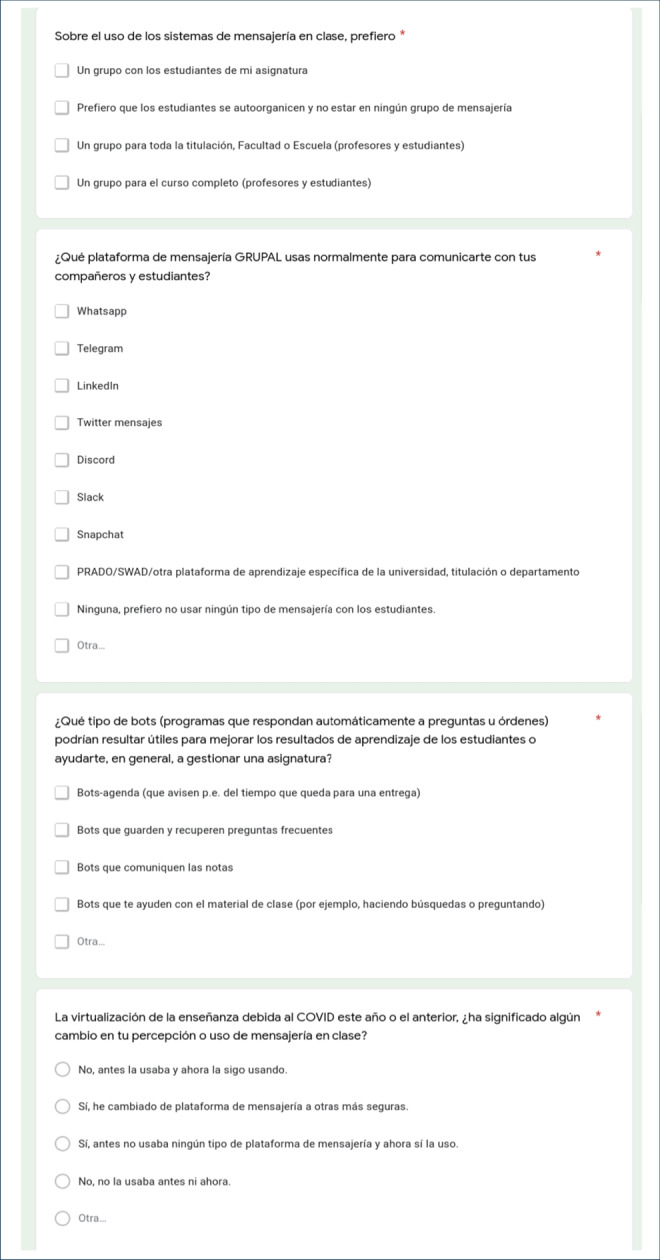
Screen capture of the Spanish-language form used for the **first survey**

**Fig. 19:**
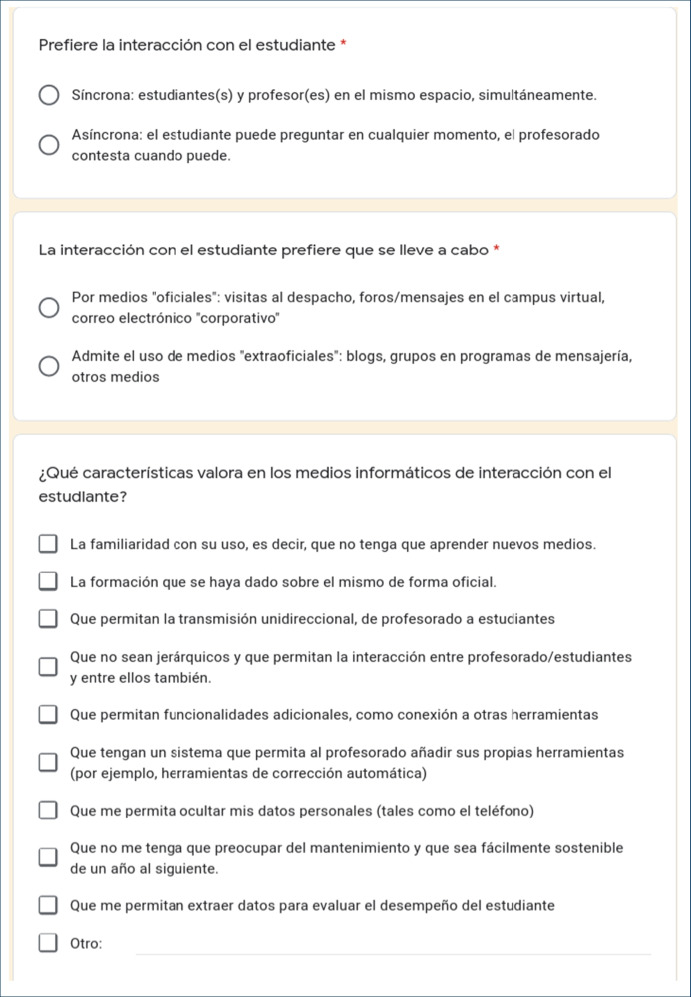
Screen capture of the Spanish-language form used for the **second survey**

## Data Availability

The datasets generated and analysed during the current study are available in the edubots-paper repository, accessible from https://github.com/JJ/edubots-paper/tree/main/data.

## Appendix: Survey questions

The surveys have the same common initial demographic questions:

- Degrees or titles for which the educator was teaching, either from a university or other tertiary education institution.
- Discipline: Engineering, Social Sciences, Health and Bioscience, General Sciences, Humanities, Other.
- Gender: Man, Woman, Rather not say.
- Age: 25-35, 35-45, 45-55, more than 55.
- Experience on the job: up to 5 years, 5-15 years, 15-25 years, more than 25 years.
- Type of degree: university, other tertiary education. This question was implicit in the first survey, as there were two different versions of the survey. However, it was an explicit question in the second survey.

The **f**irst survey, shown in its original form in Fig. 18, which focused on the use of messaging apps in class, and that was used for addressing RQ1, RQ2, and RQ3, includes the following questions:

1. Regarding the use of messaging apps in class, you prefer...
  - A chat group with the students in the same course
  - Students self-organize and the teacher is not part of any chat group
  - A chat group with all the students and teachers of the School/High-School/Faculty
  - A chat group with all the students and teachers of the same study year
2. Which messaging app platform do you use to communicate with your peers or students?
  - WhatsApp
  - Telegram
  - LinkedIn
  - Twitter messages
  - Discord
  - Slack
  - Snapchat
  - The platform provided by your own institution
  - None, I do not use any messaging app to communicate with my students
3. Which kind of chatbots (software that automatically responds to questions or commands) could be useful to improve your students’ learning results or help you managing your course?
  - Agenda bots that e.g. remind students project deadlines
  - Bots that collect and provide answers to frequent asked questions
  - Bots that inform students about their grades
  - Bots that help with the class materials (e.g. searching for topics about a concept, or asking about them)

*4. Regarding the virtualization of teaching due to the COVID-19 pandemic during this year and the previous one, did it mean any change in your viewpoint or use of messaging apps in class?*

- No, I did use it before and use it now
- Yes, I started using safer alternatives
- Yes, I did not use any messaging platform before but I do use it now
- No, I have not used any messaging platform before or currently

The **second survey**, shown in its original form in Fig. 19, followed the same paths, only in this case there was a single questionnaire, and with a different scope, focusing on the kind of interaction media features that teachers value the most. It was filled by teachers that were also students in a Professional Training Course (on the use of new technologies in higher education, around 1/4 of them) as well as University of Granada teachers who knew about it by emails from the authors, or from the vicedeanship for International Relations that included it in its newsletter. This means that there might be a higher proportion of 1) teachers with few years of experience 2) teachers from the computer science faculty and 3) teachers from Granada. We think, however that there is no explicit bias in this selection, although of course specific percentages will vary.

Specifically, this survey asked the teachers, in addition to general information (such as gender, age, experience, etc), what their past experiences and needs are with regard to the use of instant messaging applications with students, and even using bots or chatbots in the classroom. In addition, the survey asked teachers about the most relevant *interaction media features* of the potential tool to be used in class.

The specific questions included in this second survey are listed below:

1. You prefer the interaction with your students to be...
  - Synchronous: students and teacher/s in the same space, simultaneously. [Synchrony - Yes]
  - Asynchronous: the student asks at anytime, the teacher responds whenever possible. [Synchrony - No]
2. Regarding the interaction with your students, you...
  - Prefer it to be done by institutional means/tools: office hours, forum/messages via the virtual campus, institutional email. [Officiality - Yes]
  - Admit the use of other means/tools: blogs, chat groups in messaging platforms, any others. [Officiality - No]
3. Which media features do you value the most for your interaction with your students?
  - Familiarity with its use i.e. that you do not need to learn a new tool. [Familiarity]
  - The official training provided by your institution on its use. [Official formation]
  - That only one-directional communication is allowed (from teachers to students). [Unidirectionality]
  - Non-hierarchical tools that allowed teacher-to-student and student-to-student communication. [Horizontally]
  - Connectivity to other tools, for instance common log in. [Connectivity]
  - Possibility for teachers to develop/add their own functionalities (e.g. automatic correction tools). [Pluggability]
  - Possibility for users to hide personal data (such as your telephone number). [Hidden Phone Number]
  - Not having to worry about maintenance and that it is easily sustainable from year to year. [Sustainability]
  - That it offers the chance of extracting data to assess the student performance. [Analytics]

## Footnotes

1 “Lancaster University launch pioneering chatbot companion for students” https://www.lancaster.ac.uk/news/lancaster-university-launch-pioneering-chatbot-companion-for-students

2 In Spain, higher education institutions that would generally known as colleges in other places, are simply called high schools, and provide tertiary or associate degrees that do not have the same consideration as university degrees.

3 This **first survey** was split into two separate Google forms, one for university teachers and another for teachers in tertiary education institutions outside the university system (vocational training, including 1- and 2-year degrees). The questions and responses were the same, except for the type of tertiary degrees that were considered.

4 Original text in Spanish: ¿ El ítem está bien redactado y sin ambigüedad?

5 This addresses the issue of insecure/unsafe applications which do not encrypt messages or can be easily compromised by exposing information

6 Most university classes take place once or twice a week, as opposed to daily classes with the same teacher in other tertiary education, and hold usually twice as many persons as the typical tertiary education class

7 We seem to have one of these, at least in Spain, every 10 years or so.
